# Cost-effectiveness analysis of maternal vaccination against Group B streptococcus in Japan

**DOI:** 10.1016/j.jvacx.2023.100332

**Published:** 2023-06-16

**Authors:** Sumire Sorano, Simon R Procter, Anna C Seale

**Affiliations:** a Department of Disease Control, Faculty of Infectious and Tropical Diseases, London School of Hygiene & Tropical Medicine, United Kingdom; b School of Tropical Medicine & Global Health, Nagasaki University, Japan; c Department of Infectious Disease Epidemiology, Faculty of Epidemiology and Population Health, London School of Hygiene & Tropical Medicine, United Kingdom; d Warwick Medical School, University of Warwick, United Kingdom

**Keywords:** Group B streptococcus (GBS), Vaccine, Maternal immunization, Cost-effectiveness analysis, Neonatal sepsis, Neonatal meningitis

## Abstract

**Background:**

Group B Streptococcus (GBS) is a leading pathogen causing life-threatening bacterial infections in neonates (early- or late-onset) and infants, and is associated with preterm and stillbirth. Japan introduced national guidelines to reduce early-onset neonatal GBS disease, with universal prenatal screening and intrapartum antimicrobial prophylaxis (IAP). However, screening/IAP does not prevent GBS associated late-onset disease, preterm or stillbirth. Maternal GBS vaccines in development are targeted at infant GBS disease but may provide benefit across perinatal outcomes. We aimed to assess cost-effectiveness of a future maternal GBS vaccine, for a base case prevention of infant GBS disease in combination with screening/IAP compared to screening/IAP alone.

**Methods:**

We used a decision tree model to estimate cases of infant GBS disease, deaths, and neuro-developmental impairment (NDI), GBS-related stillbirths, and the associated costs and loss in Quality-Adjusted Life Years (QALYs). We calculate the threshold price at which a vaccine would be cost-effective assuming a cost-effectiveness threshold of ¥5 million/QALY. We explored the potential benefit of a maternal GBS vaccine that also prevents preterm birth in a scenario analysis.

**Results:**

Maternal GBS vaccination in Japan could prevent an additional 142 infant GBS cases annually, including 5 deaths and 21 cases of NDI, and 13 stillbirths compared to screening/IAP alone. The incremental cost-effectiveness ratio (ICER) was ¥3.78 million/QALY with a vaccine cost of ¥5,000/dose. If the QALY lost for stillbirth is included, the ICER is reduced to ¥1.78 million/QALY. Median threshold vaccine price was ¥6,900 per dose (95 % uncertainty interval ¥5,100 to ¥9,200 per dose). If maternal GBS vaccination also prevented half of GBS-associated preterm, the ICER would be reduced to ¥1.88 million/QALY.

**Conclusions:**

An effective maternal GBS vaccine is likely to be considered cost-effective in Japan at a price of ¥5,000/dose. Effectiveness against other adverse perinatal outcomes would increase health benefits and cost-effectiveness.

## Introduction

Group B Streptococcus (GBS; *Streptococcus agalactiae*) is one of the most common pathogens causing stillbirth and life-threatening bacterial infections in neonates and infants worldwide. A systematic review reports that GBS is associated with 1 % (95 % confidence interval [CI]: 0–2 %) of all stillbirths in developed countries and 4 % (95 % CI: 2–6 %) in Africa [1]. Infant invasive GBS disease is divided into early-onset disease (EOD, days 0–6), and late-onset disease (LOD, days 7–89) and can present as meningitis, pneumonia, and/or sepsis without apparent focus of infection. Many countries have implemented intrapartum antimicrobial prophylaxis (IAP) to prevent early-onset GBS disease in neonates, giving intravenous antibiotics (usually penicillin–based) to mothers in labour based on either on microbiological screening or on clinical risk factors [2].

Incidence of infant invasive GBS disease differs worldwide but is subject to challenges in case ascertainment. There is also some variation in the prevalence of maternal rectovaginal GBS colonization, and variation in health system policies, with implementation of IAP based on screening or clinical risk factors [3], [4]. A recent systematic review and meta-analysis estimated overall incidence of infant GBS disease to be 0.49/1000 live births, highest in Africa (1.12/1000 live births) and lowest in Asia (0.30/1000 live births) [5].

In Japan, nationwide surveillance suggests a relatively low incidence of infant GBS disease; EOD incidence is 0.09/1000 live births and LOD incidence is 0.21/1000 live births in 2016–2020 [6] with gradual increasing trend in LOD incidence [6], [7]. The mortality and morbidity remain important: the case fatality risks of EOD and LOD are 6.5 % and 3.0 % respectively, and a significant proportion of survivors have short- and long-term consequences [6], [7]. Neonatal meningitis due to GBS has a high risk of neurodevelopmental impairment (NDI) [6], [7], with major health implications for the individual, but also cost to families, health systems and society. A national cohort study in Denmark and the Netherlands showed a history of infant GBS disease was associated with more clinic visits and hospital admissions [8].

Japan introduced national guidelines for universal prenatal screening and IAP to prevent early onset GBS disease in 2008, and updated these guidelines in 2011, 2014, 2017 and 2020 [9]. The current maternal GBS guideline recommends prenatal screening at 35–37 weeks’ gestation using cultures from the vagina and rectum, and administration of IAP in labour for women with positive GBS culture or with an unknown culture result [9]. However, screening strategies are limited; maternal GBS colonization can vary and there can be inconsistencies between culture results in the late third trimester and at the time of delivery as well as imperfect sensitivity (sensitivity 86.6 %, specificity 96.0 %) [10]. In addition, IAP does not aim to prevent LOD, in infants, which poses a substantial burden [11], and when given in labour IAP cannot prevent preterm birth and/or stillbirth. Other limitations include potential risk of antimicrobial resistance associated with frequent antibiotic use [12], [13].

In addition to the current strategy of IAP, maternal GBS vaccination could further reduce the burden of infant GBS disease through transplacental transfer of protective antibodies. Different types of GBS vaccines are under development including multivalent polysaccharide-based vaccines and protein-based vaccines [14]. The World Health Organization (WHO) specify in their preferred product characteristics that a maternal GBS vaccine should cover the diversity of bacterial capsular types or target protein expression prevalence and polymorphism, targeting at least 90 % of the current invasive disease isolates [15]. In terms of capsular types, although there is some geographical variation, serotype III accounts for the majority (61.5 %) of invasive infant GBS disease worldwide with 97 % of cases caused by five serotypes (Ia, Ib, II, III, and V) [5]. The pattern is similar in Japan, where serotypes III (57.9 %), Ia (21.9 %), and Ib (11.7 %) predominate and five serotypes (serotypes Ia, Ib, II, III and V) are responsible for 96.4 % of pathogens identified [6].

A maternal GBS vaccine aims to prevent GBS-related stillbirth and invasive infant disease (EOD and LOD) [16], but may also reduce GBS-associated preterm birth. Cost-effectiveness analyses of GBS vaccine in some countries (such as the UK, USA and South Africa) and regions (sub-Saharan Africa) have suggested maternal GBS vaccination may be cost-effective [17], [18], [19], [20], [21], [22]. However, this has not been assessed in Japan, where GBS disease incidence, and the health system, are different.

Under the Japanese healthcare system, fees for health services and pharmaceuticals are set by the Central Social Insurance Medical Council (Chuikyo) as part of the Japanese Ministry of Health, Labor and Welfare and all healthcare providers throughout Japan are required to comply with the fee and calculation requirements [23]. In order to contain the rising healthcare expenditure, Japan introduced Health Technology Assessment (HTA) in 2016, where fees in the insurance system are adjusted based on evidence from cost-effectiveness analysis for selected pharmaceuticals and medical devices after approval [24].

The aim of this article is to assess the potential cost-effectiveness of introducing maternal GBS vaccination alongside current screening and IAP practice in Japan. This includes a threshold analysis to determine the maximum price at which a vaccine is likely to be cost-effective in Japan, and a scenario with protection against wider adverse perinatal outcomes associated with GBS-related preterm birth.

## Materials and methods

### Decision problem

The decision problem concerns all pregnant women (based on the birth statistics in 2019) [25], offered a GBS hexavalent vaccine in pregnancy, with screening-based IAP, compared to screening-based IAP alone, in Japan. The time horizon was over a lifetime, with outcomes including: number of cases of infant GBS disease (EOD and LOD), impairment and deaths from GBS disease averted, GBS-related stillbirth averted, incremental cost, incremental life-year, incremental Quality-Adjusted Life Year (QALY), cost per life-year gained, cost per QALY gained. Currently, there is no universally accepted way to value QALY loss due to stillbirth. As a baseline, we did not include QALY loss of the foetuses who died before the birth. However, we conducted alternative analysis where we fully value the QALYs of stillborn babies. We also assessed the threshold vaccine cost. In a scenario analysis we further assessed the potential benefit of a maternal GBS vaccine effective against GBS-associated preterm birth [26], which were not included in the base case scenario because of limited evidence for these outcomes. We performed our analysis in the context of the Japanese healthcare system, following the Japanese guidelines of Cost Effectiveness Evaluation [27]. Costs and health benefits were assessed over the lifetime of the children born to mothers given the maternal GBS vaccine or not. Table 1 shows a summary of decision problem.

**Table 1:** Summary of the decision problem for a maternal Group B Streptococcal (GBS) vaccine cost-effectiveness analysis in Japan.

**Population**	All pregnant women at least 22 weeks of gestation in Japan
**Intervention**	Offering hexavalent GBS vaccination to all pregnant women of 22 weeks of gestation and onward in Japan. Screening-based intrapartum antibiotic prophylaxis (IAP) will be continued.
**Comparator**	Current prevention strategy (screening-based IAP alone)
**Cost perspective**	Healthcare system perspective
**Time horizon**	Lifetime from birth
**Form of evaluation**	Life-table approach and year-long cycles
**Discount rate**	2.0 % per year
**Outcomes**	Number of infant GBS disease averted Number of NDI from GBS disease averted Number of deaths from GBS disease averted Number of GBS-related stillbirth averted Incremental cost Incremental life-year Incremental Quality Adjusted Life Year (QALY) Cost per life-year gained Cost per QALY gained Threshold vaccine price to be cost-effective

### Vaccine

A hexavalent GBS vaccine is currently in development and covers the widest range of serotypes, (Ia, Ib, II, III IV and V) accounting for 98.8 % of infant invasive GBS disease in Japan [6], [7]. The WHO recommend one dose regimen in the second or third trimester of pregnancy in their preferred product characteristics (PPCs) document [16]. Considering the limit of viability being 22 weeks of gestation in Japan, and the primary purpose of the vaccine to prevent GBS-related stillbirth and GBS EOD and LOD disease, the target of vaccination was assumed to be pregnant women of at least 22 weeks’ gestation. We assumed vaccine efficacy (VE) to be consistent regardless of the timing of birth in the baseline analysis. However, as some studies suggest that placental transfer of IgG might differ depending on gestational age, we conducted a sensitivity analysis changing VE for preterm vs term birth.

### Model structure

**Fig. 1:**
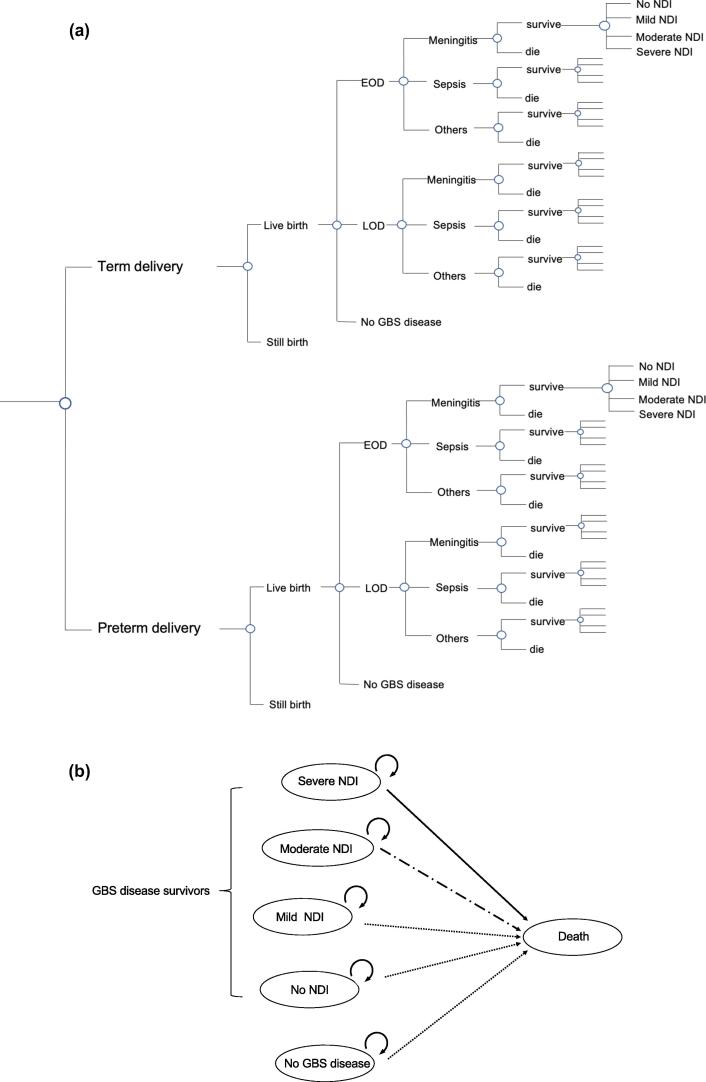
A decision tree model and an embedded Markov model for calculating the lifetime pay-offs of a maternal Group B Streptococcal (GBS) vaccine in Japan. a. Diagram of decision tree model. b. An embedded Markov model for calculating the lifetime pay-offs of a maternal Group B Streptococcal (GBS) vaccine. Fig. 1. (a) Diagram of decision tree model for base case scenario. Timing of birth (term vs preterm) and probability of live birth vs stillbirth, risk of infant GBS disease and the consequences in each case are integrated in the decision tree. Infant GBS disease was divided into early onset disease (EOD) and late onset disease (LOD) with different probabilities of death and neuro-developmental impairment (NDI). (b) Survivors of GBS disease are classified into four categories of NDI: severe, moderate, mild or none. Severity of NDI is assumed fixed over life, without transition from one category to another. Those who did not have GBS disease and those with mild or no NDI have age-dependent risk of death whereas for those with severe and moderate NDI have increased age-dependent risks of death.

We developed a decision tree model for infant GBS disease (EOD and LOD) and health outcomes, including death and long-term NDI (Fig. 1a). We incorporated the timing of delivery (preterm vs term) into the model because of the differing risk of GBS-related stillbirth, incidence and prognosis of GBS-diseases. Infant GBS disease was categorised by time of onset (EOD and LOD) and type of infection (meningitis, sepsis and others), each with specific risks of death and NDI.

We used an embedded Markov model for calculating the lifetime life-years and QALYs (Fig. 1b). Survivors of GBS disease are classified into four states based on severity of NDI: severe, moderate, mild or no NDI. Severity is assumed to be fixed over a life time. A static model was chosen because of limited evidence [28], for vaccination on maternal GBS transmission or colonization.

### Parameters – Disease outcome

Table 2 summarises the parameters used in the simulation. Incidence of infant GBS disease was informed by the latest nationwide survey in Japan [7]. Frequency of types of GBS disease and the case fatality risk were informed by this and the previous nationwide survey [[6], [7]]. Frequency of NDI was parametrised based on this survey, and another Japanese study reporting 41 cases of GBS meningitis in 1996 [29] and, because of limited data from within Japan, studies outside of Japan [30] (supplemental file, section 1). Those without GBS disease, and those with mild or no NDI were assumed to have an age-dependent risk of death, derived from Japanese life table 2020 [31] whereas those with severe and moderate NDI were presumed to have elevated age-dependent risks of death, modelled based on data on CP (supplemental file, section 2).

**Table 2:** Base case parameter values, ranges for deterministic analysis and parameter distributions for probabilistic sensitivity analysis of a maternal Group B Streptococcal vaccine in Japan.

**Parameter**	**Base value**	**Range**	**Distribution used in PSA**	**Source**
GBS disease incidence				
**Term deliveries**				
EOD incidence (per 1000 live births)	0.07	0.03–0.19	Beta(147,1950706)	6,7
LOD incidence (per 1000 live births)	0.17	0.08–0.37	Beta(472,2822681)	6,7
EOD; proportion of meningitis	0.294	0.241–0.352	Dirichlet(75, 176, 4)	6,7
sepsis	0.694	0.635–0.743		
others	0.012	0.013–0.016		
LOD; proportion of meningitis	0.433	0.397–0.470	Dirichlet (289,352,27)	6,7
sepsis	0.528	0.483–0.566		
others	0.039	0.037–0.047		
Risk of death: meningitis	0.047	0.030–0.074	Beta(18,346)	6,7
sepsis	0.013	0.007–0.027	Beta(8,520)	
others	0.000	0.000–0.116	Beta(1,30)	
**Preterm deliveries**				
EOD incidence (per 1000 live births)	0.34	0.15–0.88	Beta(41,115962)	6,7
LOD incidence (per 1000 live births)	0.92	0.44–2.02	Beta(156,167797)	6,7
EOD; proportion of meningitis	0.149	0.081–0.241	Dirichlet(11, 58, 1)	6,7
sepsis	0.851	0.725–0.904		
others	0.016	0.016–0.035		
LOD; proportion of meningitis	0.261	0.260–0.376	Dirichlet (82, 168, 7)	6,7
sepsis	0.714	0.599–0.716		
others	0.026	0.023–0.027		
Risk of death: meningitis	0.098	0.023–0.136	Beta(5, 68)	6,7
sepsis	0.056	0.056–0.144	Beta(23, 203)	
others	0.000	0.000–0.410	Beta(1,7)	
Proportion of NDI among survivors of meningitis Mild NDI Moderate NDI Severe NDI	0.126 0.139 0.055	0.081–0.189 0.092–0.204 0.029–0.104	Beta(19.48, 129.52) Beta(21.43, 127.57) Beta(9.08, 139.92)	7,29,30
Proportion of NDI among survivors of sepsis Mild NDI Moderate NDI Severe NDI	0.026 0.028 0.011	0.012–0.055 0.014–0.059 0.004–0.035	Beta(6.89, 224.11) Beta(7.52, 223.48) Beta(3.58, 227.42)	6,7
Proportion of NDI among survivors of others Mild NDI Moderate NDI Severe NDI	0.003 0.003 0.001	0.002–0.232 0.002–0.232 0.002–0.232	Beta(1, 14) Beta(1, 14) Beta(1, 14)	6,7
Livebirths (annual)	865,239			25
Stillbirth >= 22 weeks GA (annual)	2377			25
Days of hospital stay (NICU/GCU) Meningitis Sepsis Others	21 12 10	11–35 6–20 5–17	Gamma(11.1, 1.89) Gamma(11.1, 1.08) Gamma(11.1, 0.9)	s14-18
Cost of per day: NICU	¥105,390	52,826–175,802	Gamma(11.1, 9485.1)	34
Cost of per day: GCU	¥56,970	28,556–95,032	Gamma(11.1, 5127.3)	34
Annual long-term cost Severe Moderate Mild	¥1,647,020 ¥167,378 ¥16,000	825,554––2,747,406 83,897––279,205 8,020––26,690	Gamma(11.1, 148231.8) 34 Gamma(11.1, 15064.0) s21 Gamma(11.1, 1440) 35	
HR-QOL NDI cases Severe Moderate Mild	0.595 0.782 0.954	0.47–0.72 0.62–0.944 0.91–0.998	Uniform(0.47, 0.72) Uniform(0.62, 0.944) Uniform(0.91, 0.998)	17, s10-12
Vaccination cost per dose Vaccine price Administration cost	5000 4000 1000	1200–10000 1000–7000 200–3000		33
Vaccination coverage	0.70	0.50–0.90	Uniform(0.60–1.00)	
Vaccination efficacy	0.80	0.60–1.00	Uniform(0.60–1.00)	16
Vaccine strain coverage (hexavalent)	0.988			6,7
Proportion of GBS-related stillbirth	0.01	0–0.02	Uniform (0–0.02)	1
For additional analysis:				
Screening based on culture	¥1,700			34
IAP (intravenous ampicillin 2 g + 1 g)	¥1,525			34

PSA = Probabilistic Sensitivity Analysis, EOD = Early Onset Disease, LOD = late onset disease, GA = Gestational Age, NICU = Neonatal Intensive Care Unit, GCU = Growth Care Unit, NDI = neurodevelopmental impairment, Small “s” before the source number indicates source in supplemental file.

There were no data on health-related quality of life (HR-QOL) of GBS disease NDI among Japanese people. Even globally there was no HR-QOL data for infant GBS disease survivors except for unpublished data cited in previous cost-effective analyses in UK and The Netherlands.[17], [32] We therefore assumed health state utilities for severe NDI based on severe mental retardation, severe cerebral palsy with Gross Motor Function Classification System (GMFCS) level III-V, moderate NDI based on mild mental retardation, moderate cerebral palsy with Gross Motor Function Classification System (GMFCS) level I-II, mild NDI based on mild hearing impairment, Attention-Deficit/Hyperactive Disorder (ADHD)) (supplemental file, section 3).

### Parameters - cost

Since a vaccine is not currently licensed no information is currently available on vaccine price. We assumed that the vaccine price would be similar to recent new vaccines in Japan, in the range ¥1000-7000 [33]. The fee for a single injection is ¥200 for visits covered by public insurance set by Ministry of Health, Labor and Welfare [34]. For visits not covered by public insurance, vaccine administration (including consultation and injection procedure) is around ¥3000 [33]. We assumed that a maternal GBS vaccine would be administered during regular antenatal care, without requiring additional visits, and we assumed the administration cost to be between these costs.

Acute hospitalization cost per day for infants was informed by the fee for health services set by Ministry of Health, Labor and Welfare [34] (supplemental file, section 4). Length of hospital stay was parametrized based on reports in the Japanese literature and the recommendation in Japanese clinical guideline (supplemental file, section 5).

Long-term healthcare cost due to NDI was assumed to be constant over life. It was calculated based on (1) the fee for health services set by Ministry of Health, Labor and Welfare [34] and (2) a survey on Medical Care Benefit by National Health Insurance [35] which reports amount of healthcare fee spent by diagnosis, service categories (inpatient, outpatient, pharmaceutical, dental care), age groups etc (supplemental file, section 6). Costs for screening and IAP were calculated based on the fee for health services set by Ministry of Health, Labor and Welfare (supplemental file, section 7) [34]. All costs were calculated in 2021 Japanese yen (¥).

### Analysis

The incremental cost-effectiveness ratio (ICER) is calculated as the incremental cost per QALY gained. A threshold analysis was conducted to determine the maximum price at which a vaccine is likely to be cost-effective in Japan.

A survey in Japan showed that ¥5 million (38,000USD)/QALY is the willingness-to-pay (WTP) threshold in Japan [36]. Also, under recently adopted HTA guidelines, interventions with an ICER above ¥5 million/QALY gained became the target for price adjustment in Japan [24]. Therefore, we assumed an ICER of ¥5 million/QALY gained as the cost-effectiveness threshold. Baseline vaccine efficacy was assumed to be 80 %, based on the WHO preferred product characteristics [15].

### Deterministic sensitivity analysis

One-way sensitivity analysis was performed to examine the impact of uncertainty in individual parameter values on the resulting cost-effectiveness. Two-way sensitivity analysis was conducted to explore the combination of vaccine efficacy and vaccine price.

### Scenario analysis; preterm birth and stillbirth

We explored the potential benefit of maternal GBS vaccination to prevent GBS-related preterm birth in a scenario analysis. An estimated risk ratio (RR) of preterm birth was reported to be 1.21 (95 % CI: 0.99–1.48; P = 0.061) in women with positive GBS colonisation in another worldwide systematic review [26]. We estimated the number of GBS-related preterm births in Japan from those data (supplementary file, section 8), and conducted scenario analysis assuming vaccine efficacy of 50 % and 80 % against GBS-related preterm births. For preterm birth, distribution of gestational age at delivery is given by Japan Perinatal Registry which also provides neonatal mortality according to gestational week at birth [37]. Cost for preterm care as well as neonatal death averted were considered.

The analysis was conducted with R version 4.0.3 using standard packages and graphs were plotted with Microsoft Excel.

### Discount rate

Following Japanese guidelines, both future costs and health outcomes were discounted at 2.0 % per year [27]. We conducted a sensitivity analysis with discount rates for health and for cost varied separately from 0 % to 5.0 %.

### Probabilistic sensitivity analysis (PSA)

A probabilistic sensitivity analysis (PSA) was carried out using 1000 Monte Carlo simulations (parameters and distributions detailed in Table 2). Detailed parametrisation is explained in supplementary file, section 9. In brief, beta distribution and Dirichlet distribution were used for probabilities of two outcomes and three outcomes respectively. We used a gamma distribution for cost and length of hospital stay because distribution of costs/resource tend to be right skewed. For parameter ranges, hospitalization cost per day, length of stay, and annual long-term costs were assumed to have a standard deviation which is 30 % of the baseline value.

We explored the maximum vaccine price for a maternal GBS vaccine to be cost effective both in deterministic and probabilistic analysis for infant GBS disease. For probabilistic analysis, we estimated per-dose vaccine costs at which 97.5 %, 50.0 % and 2.5 % of iterations fall under the cost-effective threshold of ¥ 5 million/QALY gained.

## Results

### Deterministic model results

We estimated a maternal GBS vaccination would prevent an additional 142 infant GBS cases annually, including 5 deaths and 21 cases with NDI using an assumption of 70 % maternal GBS vaccine coverage and 80 % vaccine efficacy against infant invasive GBS disease. In the baseline analysis where QALYs for stillbirth are not included, 325 life years and 483 QALYs would be gained at an incremental cost to the health care system of ¥ 1,823 million, resulting in an ICER of ¥ 3.78 million/QALY. If QALYs and LYs lost for stillbirth are included, in total of 866 life years and 1023 QALYs would be gained, resulting in an ICER of ¥ 1.78 million/QALY. The results of the base case scenario are summarised in Table 3.

**Table 3:** Results of the base case scenario of cost-effectiveness analysis of a maternal Group B Streptococcal vaccine in Japan.

	**Current strategy**	**Proposed strategy**	Difference
	Screening-based intrapartum antibiotic prophylaxis (IAP) alone	Vaccination with screening-based intrapartum antibiotic prophylaxis (IAP)	
Infant disease cases	257.1	114.9	−142.3
NDI cases	37.6	16.8	−20.8
Infant deaths	9.0	4.0	−5.0
GBS-related stillbirth	23.8	10.6	−13.2
GBS-related preterm birth	1468.1	1468.1	0
Life Years gained	35,578,224	35,578,548	325
		(35,579,089)	(8 6 6)
Quality Adjusted Life Years (QALY) gained	35,577,938	35,578,421	483
		(35,578,962)	(1023)
Costs (in million)	¥5,330	¥ 7,153	¥ 1,823
Maternal immunisation	0	¥ 3,037	¥ 3,037
Short-term cost	¥1,795	¥ 802	¥ −993
Long-term cost	¥399	¥ 178	¥ −221
Screening & IAP cost	¥1,668	¥1,668	0
Cost for GBS-related preterm birth	¥1,468	¥1,468	0
Cost per QALY gained		¥ 3.78 million/QALY	
		(¥ 1.78 million/QALY)	
Cost per life-year gained		¥ 5.61 million/LY	
		(¥2.11 million/LY)	
Cost per case prevented	¥ 12.81 million/case		
Cost per death averted	¥ 364.67 million/death		
Threshold vaccine cost per dose*	¥ 5,900 per dose		
	(¥10,400 per dose)		

In the baseline analysis, QALYs loss due to stillbirth are not included. Alternatively, the values in () is where QALYs of stillborn babies are fully valued.

* Maximum vaccination cost (including vaccine price and administration cost) for ICER to be < ¥5 million/QALY with deterministic approach.

The maximum vaccine price for maternal GBS vaccination to be cost-effective at threshold of ¥5 million/QALY under the base case scenario was ¥ 5,900 per dose whereas it was ¥10,400 per dose when QALYs lost for stillbirth were included.

### One-way sensitivity analysis

**Fig. 2:**
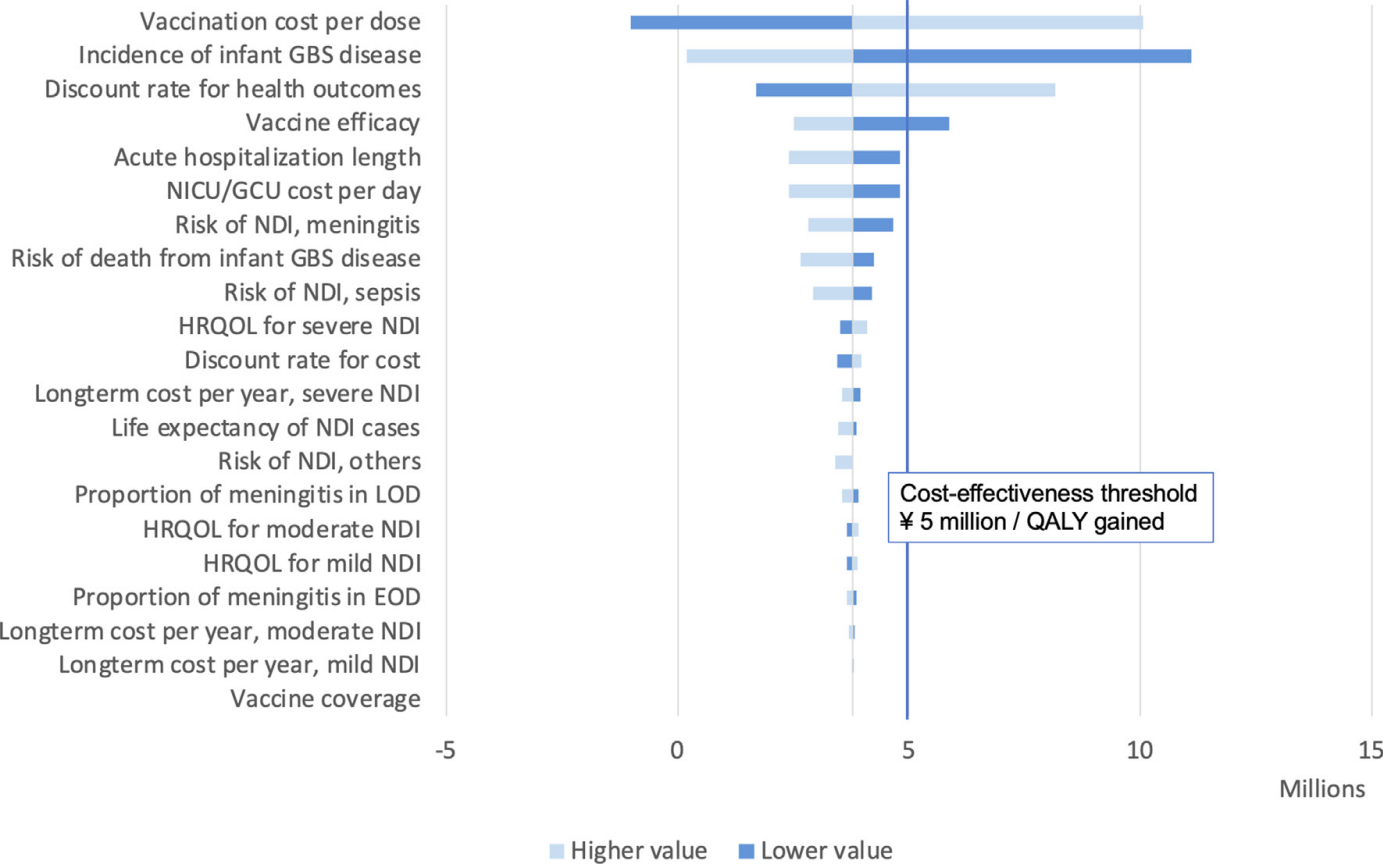
Tornado graph of one-way sensitivity analysis of cost-effectiveness analysis of a maternal Group B Streptococcal vaccine in Japan.

Variables influencing the results of cost-effectiveness using a one-way sensitivity analysis were vaccine price, disease incidence and vaccine efficacy (Fig. 2). The results of the cost-effectiveness analyses were highly sensitive to the discount rate for health outcomes, whereas the ICER did not differ much when varying the discount rate for cost as most costs occur in the first year.

### Two-way sensitivity analysis

**Fig. 3:**
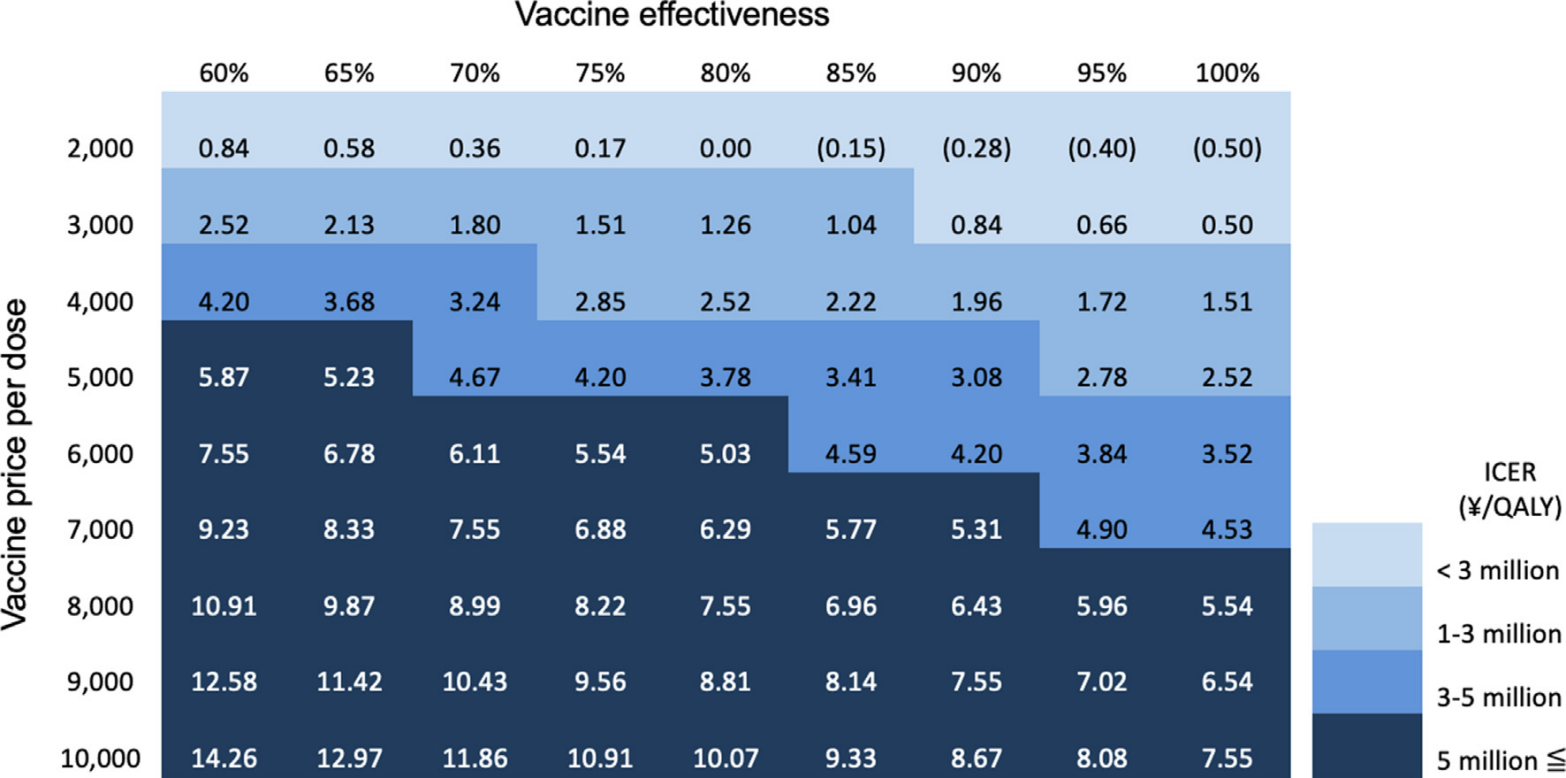
Two-way sensitivity analysis of cost-effectiveness analysis with different maternal GBS vaccination cost (including vaccine price and administration cost) and varying vaccine effectiveness in Japan.

To be cost-effective at a threshold ICER of ¥ 5 million/QALY, a maternal GBS vaccine needs to be at least 95 % effective and priced less than ¥ 7,000, or at least 60 % effective and price less than ¥ 4,000. Different ICERs are found with varying vaccine cost per dose and vaccine efficacy (two-way sensitivity analysis in Fig. 3).

### Scenario analysis

We conducted scenario analysis where maternal GBS vaccination is assumed to be less efficacious against preterm birth. If vaccine efficacy is lower for preterm birth by 20 %, infant GBS disease cases prevented would be reduced from 142.3 to 135.5 and stillbirth prevented decreased from 13.2 from 11.8, resulting in ICER from ¥ 3.78 million/QALY to ¥ 4.13 million/QALY (Table 4).

**Table 4:** Results of scenario analysis where maternal GBS vaccination is assumed to be less efficacious against preterm birth.

	Same VE for preterm and term (Baseline)	20 % reduced VE for preterm
Infant disease cases prevented NDI cases prevented Infant deaths prevented Stillbirth prevented	142.3 20.8 5.0 13.2	135.5 20.0 4.6 11.8
Incremental cost	¥ 1.82 billion	¥ 1.88 billion
Incremental QALY	483	455
ICER (million yen/QALY)	¥ 3.78 million/QALY	¥ 4.13 million/QALY
Threshold vaccination cost*	¥ 5,900	¥ 5,600

The baseline scenario assumes that vaccine efficacy (VE) is the same regardless of the timing of birth. Alternative scenario assumes that VE is reduced by 20 % for preterm birth.

* Maximum vaccination cost (including vaccine price and administration cost) for ICER to be < ¥5 million/QALY with deterministic approach.

We conducted another scenario analysis where vaccine is effective to prevent preterm birth. If a vaccine efficacy against GBS-related preterm birth is 50 % and 80 %, an estimated 725 and 1160 preterm births respectively would be prevented, saving ¥ 725 and ¥ 1160 million, and reducing the ICER to ¥ 1.88 million/QALY and ¥ 1.03 million/QALY respectively (Table 5).

**Table 5:** Results of scenario analysis where maternal GBS vaccine is effective against GBS associated preterm birth in Japan.

	No VE against PTB (Baseline)	50 % VE against PTB	80 % VE against PTB
Preterm birth prevented Preterm neonatal death prevented	0 0	725.3 2.4	1160.4 3.9
Cost for preterm birth avoided	0	¥ 725.25 million	¥1,160.40 million
Incremental cost	¥ 1.82 billion	¥ 1.10 billion	¥ 0.66 billion
Incremental QALY	483	582	642
ICER (million yen/QALY)	¥ 3.78 million	¥ 1.88 million	¥ 1.03 million
Threshold vaccination cost*	¥ 5,900	¥ 7,900	¥ 9,100

The baseline scenario assumes no effect of maternal vaccination to prevent GBS-related preterm birth. We conducted scenario analysis of 50 % and 80 % vaccine efficacy (VE) against GBS-related preterm birth.

* Maximum vaccination cost (including vaccine price and administration cost) for ICER to be < ¥5 million/QALY with deterministic approach.

### Probabilistic sensitivity analysis

**Fig. 4:**
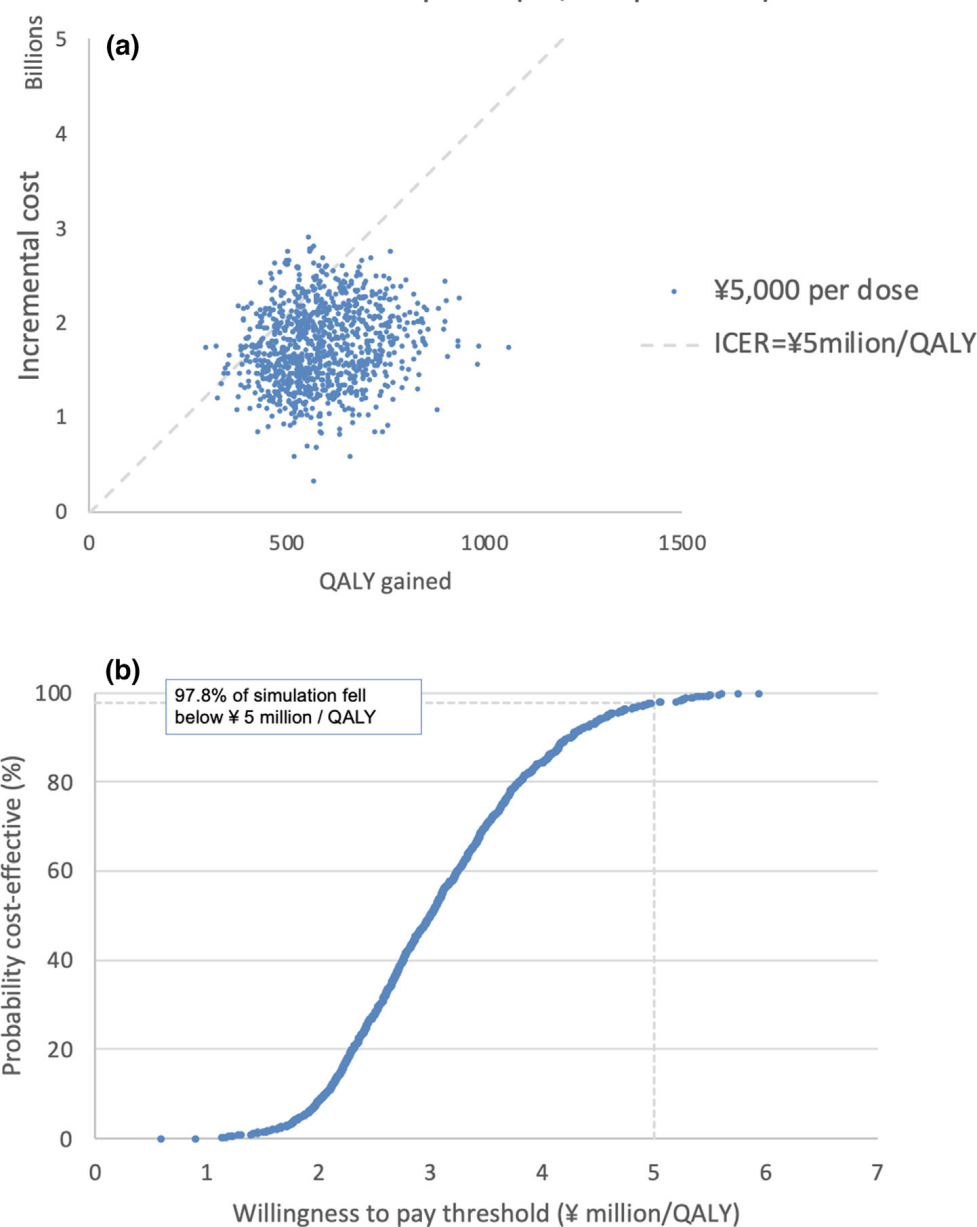
Result of probabilistic sensitivity analysis based on ¥5,000 per dose (including vaccine price and administration cost) of cost-effectiveness analysis of a maternal Group B Streptococcal vaccine in Japan.4a. Cost-effectiveness plane based on ¥5,000 per dose. Monte Carlo probabilistic sensitivity analysis of 1000 iterations, for base case scenario. The incremental cost (¥) of the maternal vaccination strategy with screening-based IAP comparing with that of screening-based IAP alone is plotted in the y axis, with the x  axis displaying the incremental QALYs gained. Of the 1000 iterations 97.8 % fell under the ¥5 million threshold of cost per QALY gained. 4b. Cost-effectiveness acceptability curves based on ¥5,000 per dose (including vaccine price and administration cost).

Fig. 4 shows the result of the probabilistic sensitivity analysis with Monte Carlo iterations under assumptions of vaccine prices of ¥5000 per dose. At a threshold of ¥5million/QALY, 97.8 % fell under the threshold.

**Fig. 5:**
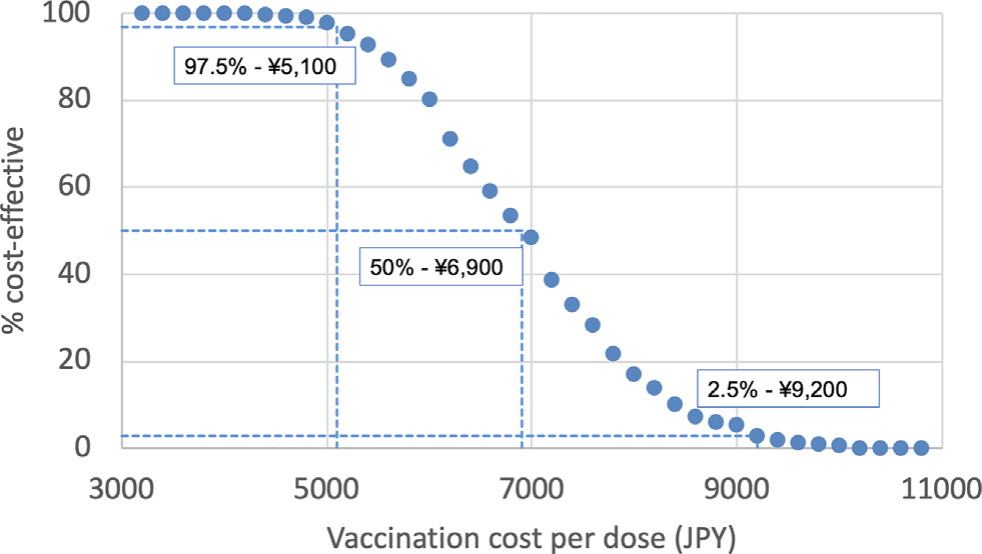
Cost-effectiveness with varying vaccination price in Japan. Percent simulations that were cost-effective (with threshold of ¥ 5 million/QALY gained) on y axis with different vaccination costs on x axis. At ¥5,100, ¥6,900, and ¥9,200 per dose, 97.5 %, 50.0 % and 2.5 % of iterations respectively fell under the cost-effective threshold of ¥5 million/QALY gained.

Fig. 5 shows percent simulations that were cost-effective (threshold of ¥ 5 million/QALY gained) under different vaccine prices. Median threshold vaccine price was ¥6,900 per dose (95 % uncertainty interval ¥5,100 to ¥9,200 per dose).

## Discussion

GBS is a leading cause of life-threatening disease in young infants, and in this first study of the cost-effectiveness of a maternal GBS vaccine in Japan, the cost per QALY based on maternal GBS vaccine price of ¥5,000 per dose, was ¥ 3.78 million/QALY, which is less than the nationally agreed level of ¥ 5 million/QALY for public health interventions. However, this cost-effectiveness analysis is highly dependent on vaccine price, disease incidence and vaccine efficacy, as was the case in the previous studies of cost-effectiveness of GBS vaccination [17], [18], [19]. Whether a maternal GBS vaccine provides benefit in terms of reducing preterm birth in addition to invasive infant GBS disease also substantially impacted cost-effectiveness.

The result of ICER was sensitive to how we value the loss from stillbirth. Currently, there is no universally accepted view of evaluating foetal outcomes, and as a baseline, we did not include QALYs of foetus lost for stillbirth. However, some argue for including foetal outcomes [38], [39]. If QALY loss of stillbirth throughout the life course are fully counted, the incremental cost per QALY based on ¥5,000 per dose will be reduced to ¥ 1.78 million/QALY. This is an area that could benefit from clarification in future HTA guidelines.

Previously conducted cost-effectiveness analysis of a maternal GBS vaccine in the United Kingdom [17], United States of America [19], [21], South African and low income settings in sub-Saharan Africa [18], [20] suggest that a maternal GBS vaccine would be cost-effective. In Japan, maternal GBS vaccination was found to be cost-effective in the base case scenario, but cost-effectiveness is sensitive to vaccine price. A survey conducted by Ministry of Health, Labor and Welfare in 2012 showed that the mean price per dose of other vaccines within the Japan immunization program ranged from ¥1195 for influenza vaccine, ¥4475 for Hib (Haemophilus influenzae type b) vaccine, ¥6773 for PCV (pneumococcal conjugate vaccine) [33]. If GBS vaccines have a similar price to PCV vaccines, then vaccination may not be cost-effective in Japan.

This is the first study we are aware of to evaluate cost-effectiveness of GBS vaccination for pregnant women in Japan. With variations in GBS incidence worldwide, we used data from a nationwide survey of infant GBS disease in Japan to parametrize our specific model, including infant GBS disease incidence, proportion of clinical syndromes, risk of neurodevelopmental impairment and death, to make the model robust and context specific. We explored a wide variety of scenarios and parameter values for sensitivity analysis. The scarcity of some data in Japanese population was, however, was a limitation, specifically HR-QOL. However, our one-way sensitivity analysis suggested overall results were not very sensitive to utility weights. Other parameters such as short-term and long-term costs with GBS disease and subsequent NDI were also uncertain, but again did not have considerable effect on the final outcome.

Another limitation, is that we used a static model because of limited evidence of potential effect of maternal vaccination to influence the dynamics of GBS transmission and colonization [28]. However, it is possible that elevated antibody titre due to vaccination might protect from acquiring GBS colonization as suggested by some studies [28]. Further research is needed to understand the potential effects of vaccination on transmission dynamics.

Finally, we did not account for maternal GBS disease burden, and spill-over effects of health impact on parents and families of children with NDI, which could increase cost-effectiveness. However, a previous study in the UK found that their cost-effectiveness analysis was insensitive to the assumption of maternal GBS disease burden [17]. In terms of spill-over effects, a survey on families with children with severe mental or physical disabilities in Kobe, Japan, showed that 88 % of main carers are mothers, and half of them had back pain, chronic fatigue and chronic lack of sleep [40]. Around a third of carers gave up working outside the home in order to care the child [40]. Considering wider societal costs such as lost productivity of parents, and inequitable impacts of women, may make a maternal GBS vaccination programme more cost-effective in Japan.

## Conclusion

At a price of ¥5,000/dose, a strategy of maternal GBS vaccination in combination with screening-based intrapartum antibiotic prophylaxis against GBS disease is likely to be cost-effectiveness in Japan. However, at higher prices vaccination may not be cost-effective, partly due to lower incidence of infant GBS disease in Japan compared to other countries where maternal GBS cost-effectiveness studies have been carried out. A vaccine that is also effective against preterm birth would increase the benefits and cost-effectiveness of a vaccine.

Improved data on the severity of NDI following invasive GBS disease, as well as evaluation of the disease impacts on families and other sectors of economy from broader societal perspective would be beneficial in assessing the case for a maternal GBS vaccine.

## Ethical approval statement

Ethical approval was not required for this study as it only uses publicly available data.

## Funding

ACS was funded by a fellowship from The Wellcome Trust (grant number 205184).

SS was funded by Nagasaki University “Doctoral Program for World-leading Innovative and Smart Education” for Global Health, KENKYU SHIDO KEIHI, by Ministry of Education, Culture, Sports, Science and Technology (MEXT) – JAPAN.

The funders have no role in the identification, design, conduct, and reporting of the analysis.

## CRediT authorship contribution statement

**Sumire Sorano:** Conceptualization, Methodology, Investigation, Data curation, Formal analysis, Software, Validation, Visualization, Writing – original draft, Writing – review & editing. **Simon R Procter:** Conceptualization, Methodology, Writing – review & editing, Supervision. **Anna C Seale:** Conceptualization, Methodology, Writing – review & editing, Supervision.

## Declaration of Competing Interest

The authors declare that they have no known competing financial interests or personal relationships that could have appeared to influence the work reported in this paper.

## Data Availability

No data was used for the research described in the article.
