# Supplementary material for: Cost-effectiveness analysis of maternal vaccination against Group B streptococcus in Japan
Source: Vaccine X. 2023 Jun 16;14:100332. doi: 10.1016/j.jvacx.2023.100332 (PMC10333677; doi:10.1016/j.jvacx.2023.100332)
Supplement: Supplementary Data 1 [file mmc1.docx]

Supplemental file

Contents

[1. Severity of Neurodevelopmental Impairment (NDI) 3](#_Toc99620404)

[2. Age-dependent risk of death for cases with severe and moderate neuro-developmental impairment (NDI) 4](#_Toc99620405)

[3. Parameters for health-related quality of life (HR-QOL) 7](#_Toc99620406)

[4. Acute care cost per day 8](#_Toc99620407)

[5. Length of hospital stay 8](#_Toc99620408)

[6. Long-term healthcare cost for cases with NDI 9](#_Toc99620409)

[7. Parameters for screening and intrapartum antibiotic prophylaxis 10](#_Toc99620410)

[8. Parameters for preterm birth 12](#_Toc99620411)

[9. Parameters and distributions used in probabilistic sensitivity analysis 15](#_Toc99620412)

[10. References 16](#_Toc99620413)

## 1. Severity of Neurodevelopmental Impairment (NDI)

Survivors of infant GBS disease are divided into four categories based on the severity of NDI: severe, moderate mild and no NDI. This followed the previous research of GBS vaccine cost-effectiveness analysis in UK^1^ where the author classified severity based on the Bayley Scales of Infant and Toddler Development, Third Edition (Bayley-III) and the second edition of the Bayley Scales tool (BSID-II).

***Severity of NDI from GBS meningitis***

Ishikawa et al.^2^ showed severe NDI (defined as severe physical and mental impairment, mostly IQ <25 and bedridden) in 5.5% (2/36) and moderate NDI (defined as one or multiple impairments of following: mental and/or physical impairment, epilepsy, hydrocephalus, auditory deficits, and visual deficits) in 13.9%(7/36) in a study which followed survivors for mean length of 2 years 11 months. This result is fairly consistent with a pooled estimate of 18% having moderate to severe NDI, in a worldwide systematic review and meta-analysis by Kohli-Lynch.^3^ Matsubara et al. reports that 32.0% (47/147) of GBS meningitis survivors had short-term NDI (at the time of hospital discharge).^4^ Therefore, we assumed that 12.6% (= 32.0% - 5.5% - 13.9%) have mild NDI. This is probably conservative considering that some mild NDI become apparent in later developmental stages (learning disability, ADHD etc).

***Severity of NDI from GBS sepsis***

Matsubara et al. showed that 6.5% (15/229) survivors of GBS sepsis had NDI, but there was no information on severity.^4^ Ree reports both increased abnormal and borderline cognitive impairment in survivors of neonatal sepsis.^5^ With limited data on severity of long-term NDI of survivors of GBS sepsis in Japan, we assumed that proportions of mild, moderate, severe NDI were the same as meningitis.

***Severity of NDI from other GBS disease***

Matsubara et al. showed that no (0/13) survivors of GBS disease other than meningitis and sepsis had short-term sequelae.^4^ Therefore, we assumed no NDI for GBS disease without sepsis or meningitis as a baseline.

## 2. Age-dependent risk of death for cases with severe and moderate neuro-developmental impairment (NDI)

Age-specific mortality of the general population (those without GBS disease, and those without NDI and with mild NDI) is given by life table of 2020^6^ in Japan. There were no data on age-dependent mortality of GBS disease survivors living with NDI. Therefore, it was substituted with data on survival among people with cerebral palsy (CP)^7^ (severe and non-severe) in Japan who had equivalent disability to our population of interest. Since survival data of CP was available only up to 20 years of age, an extrapolation of the survival curve beyond these years was needed.

Steven et al.^8^ shows three methods to project mortality rates of a particular population by adjusting mortality rates in standard table. These methods extrapolate survival curve beyond available data based on assumptions of future relationships between mortality rates in a CP cohort and standard mortality rates of general populations. Each method of adjustment is based on different assumption: the first method is based on assumption of constant excess death rate (CEDR), the second method assumes a constant relative risk (CRR), and the third assumes a proportional life expectancy (PLE). Survival curves and life expectancies calculated by each of the three methods are shown in Supplementary Figure1. The survival curve from the method of PLE falls between the other two methods and is considered to empirically perform best in the case of CP.^8^ Therefore, we used the method of proportional life expectancy (PLE) in the baseline scenario and the other two methods in sensitivity analyses.

Supplementary Figure 1. Projection of survival curve of severe Cerebral Palsy (CP) (a) and non-severe CP (b) using 3 different methods and corresponding life expectancies.


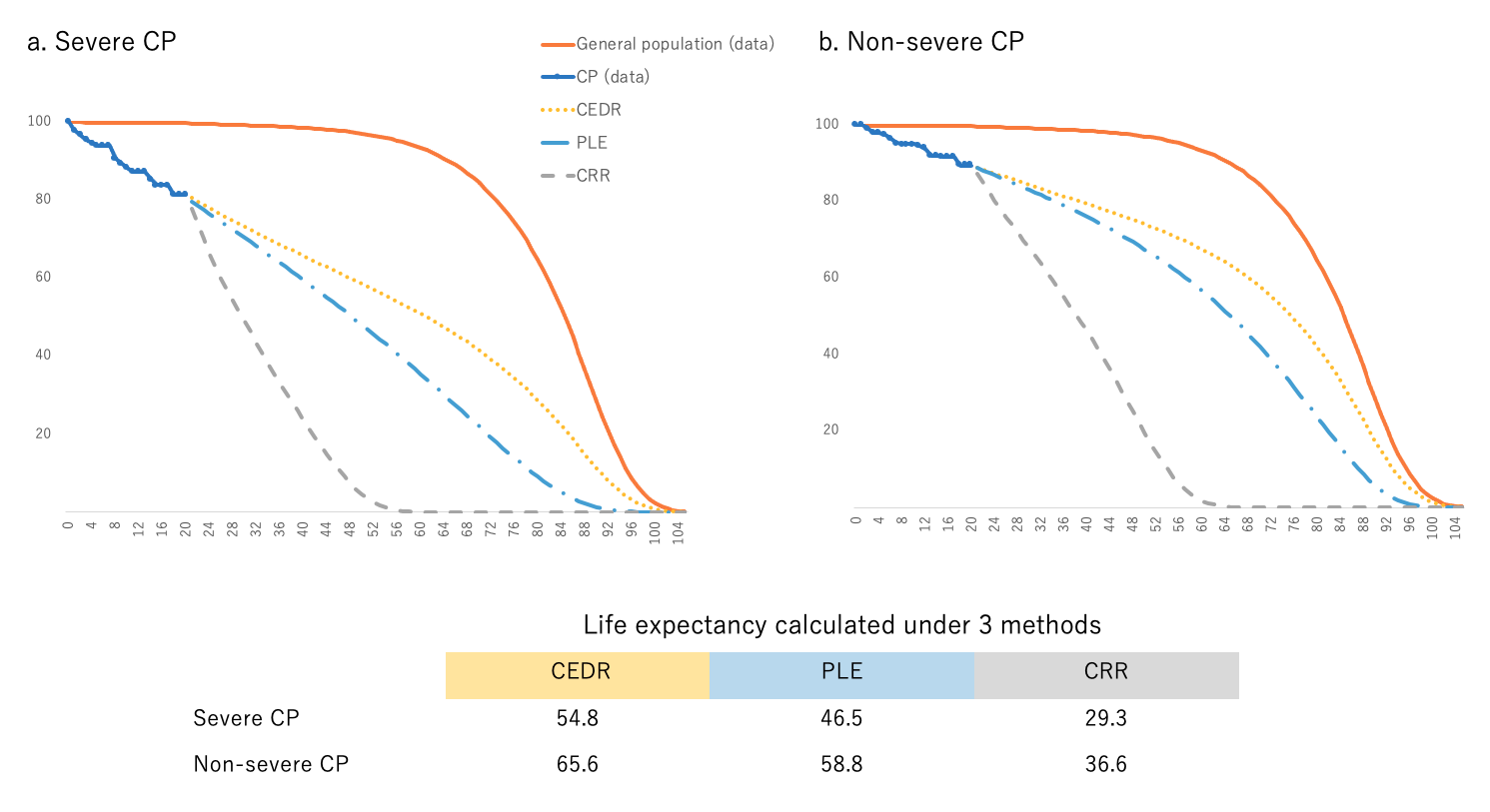


Three methods were used to project survival curve of (a) severe CP and (b) non-severe CP. Each method of adjustment is based on different assumption: the first method is based on assumption of constant excess death rate (CEDR, yellow dotted), the second method assumes constant relative risk (CRR, grey dashed), the third assumes a proportional life expectancy (PLE, blue dash dotted). Life expectancies calculated by each of the three methods are shown in the underneath.

## 3. Parameters for health-related quality of life (HR-QOL)

There were no data on health-related quality of life (HR-QOL) in people with NDI following GBS disease in Japan. Even globally there were no HR-QOL data for infant GBS disease survivors except for an unpublished study cited in a cost-effective analyses in the UK and Netherlands.^1,9^ We therefore assumed health state utilities for severe NDI based on severe mental retardation, severe cerebral palsy with Gross Motor Function Classification System (GMFCS) level III-V, moderate NDI based on mild mental retardation, moderate cerebral palsy with Gross Motor Function Classification System (GMFCS) level I-II, mild NDI based on mild hearing impairment, Attention-Deficit/Hyperactive Disorder (ADHD). A range of possible utility values for each severity was given by different studies shown in Supplementary Table 1.

We assumed that the true utility value of the GBS NDI in Japan was within these ranges and without information about the distribution of probability within these, we assumed uniform distribution for the utility values. Therefore, midpoint of each range were taken to be the baseline values.

**Supplementary Table 1 Studies used to inform HR-QOL for people with neuro-developmental impairment (NDI) from infant GBS disease.**

|  | **Severe NDI** | **Moderate NDI** | **Mild NDI** |
| --- | --- | --- | --- |
| Oostenbrink^10^ 2002  (Netherland) | 0.47 | 0.62-0.81 | 0.91 |
| Jarl^11^ 2019  (Sweden) | 0.58-0.72 | 0.77-0.89 | - |
| Carroll^12^ 2009  (US) | 0.51-0.60 | 0.79-0.84 | 0.93-0.94 |
| Heath unpublished  (cited by Giorgakoudi^1^) | 0.701 | 0.944 | 0.998 |
| Overall | 0.595 (0.47-0.72) | 0.782 (0.62-0.944) | - 1. (0.91-0.998) |

## 4. Acute care cost per day

Acute care costs for hospitalised infants are estimated as cost per diem multiplied by days in hospital. In Japan, the fee for health services is set by the Ministry of Health, Labor and Welfare (MHLW) and all healthcare providers throughout Japan are required to comply with the fee and calculation requirements. The fee for Neonatal Intensive Care Unit (NICU) per day is 105,390 yen, the fee for Growing Care Unit (GCU) per day is 56,970 yen.^13^

## 5. Length of hospital stay

Length of hospital stay was parametrized based on literature (Supplementary Table 2) as well as guidelines and consultation with Japanese neonatologists/paediatricians. We found the following two case reports^14,15^ and four case series^{Formatting Citation}^ which provided information on the length of antibiotic treatment and length of stay with infant GBS disease. As for neonatal GBS meningitis, clinical recommendation is treatment with minimum of 14-21 days of antibiotic therapy.^19^ However published work (mostly case reports) suggest a longer duration of antibiotic therapy (mostly 21-28 days) and additional 6 to 14 days of hospital stay (Supplementary Table 2). However, collection of case reports might not be representative for general GBS meningitis because of publication bias. Therefore, Japanese paediatricians were consulted who commented that antibiotics are usually given for at least 14 days for GBS meningitis and it is common practice to observe for several days after cessation of antibiotics and undergo neurological evaluation. Therefore, length of stay for meningitis was assumed to be 21 days as a baseline. Sepsis and other GBS disease would be shorter than that, so we assumed 12 days and 10 days respectively as baseline.

**Supplementary Table 2 Summary of infant GBS disease cases in literature reporting length of hospital stay**

| **Source** | **Year** | **GA at birth (weeks)** | **Sex** | **Age at onset**  **(day)** | **Infection site** | **Duration of antibiotic (day)** | **LOS**  **(day)** |
| --- | --- | --- | --- | --- | --- | --- | --- |
| Honda^14^ | 2020 | 39 | M | 22 | Meningitis, ventriculitis | 28 | 34 |
| Ueda^15^ | 2018 | 39 | M | 3 | Meningitis | 28+21(relapse) | 63 |
| Miyata^16^ | 2012 | 39 | NA | 0 | Pneumonia | NA | 11 |
|  |  | 40 | NA | 0 | Pneumonia | NA | 10 |
|  |  | 38 | NA | 5 | Sepsis | NA | 18 |
|  |  | 38 | NA | 1 | Sepsis, Pneumonia | NA | 18 |
| Matsubara^17^ | 2003 | 40 | F | 56 | Occult bacteraemia | 7 | 8 |
| Matsubara^18^ | 2000 | 40 | M | 0 | Sepsis | 1(died) | 1(died) |
|  |  | 39 | F | 0 | Sepsis | 7 | NA |
|  |  | 37 | M | 0 | Sepsis | 12 | 14 |
|  |  | 36 | F | 0 | Sepsis | 14 | NA |

GA: gestational age, LOS: length of stay, NA: Not available

## 6. Long-term healthcare cost for cases with NDI

According to the Survey on Medical Care Benefit by National Health Insurance 2017, roughly, one patient with cerebral palsy (CP) on average costed 167,378 yen (146,268 yen for inpatient care and 21,110 yen for outpatient care).^20^ However, it is not clear how the cost is distributed among patients with different severity of CP (patients with severe CP would likely incur more cost and those with mild CP would incur less). In this case, we took the average cost (167,378 yen) for patients with moderate CP.

For patients with severe NDI, the Japanese healthcare system provides nurse visits up to 3 times per week (or more for special conditions such as tracheostomy).^21^ It was assumed that each patient has a consultation with a doctor once per month, a nurse visit 3 times per week and 14 days of respite hospital stay service per year. Summing up this is an annual cost of 1,647,020 yen.

##

## 7. Parameters for screening and intrapartum antibiotic prophylaxis

Currently under screening-based intrapartum antibiotic prophylaxis (IAP), all women are offered testing for GBS at 35-37 weeks’ gestation, as well as those in preterm labour. Therefore, we assumed that the cost of screening is cost per person multiplied by number of livebirths after 22 weeks’ gestation per year, and IAP is given to those with a positive GBS test result, as well as those in preterm labour who have not had yet GBS test results.

## 8. Parameters for preterm birth

It is reported that 14.9% of pregnant women in Japan have positive GBS culture results^16^ and overall, 5.6% of all births are preterm in Japan^22^. Women with GBS colonisation have an elevated risk of preterm birth (risk ratio (RR) of 1.21 (95% CI: 0.99–1.48; P = 0.061)).^23^ From this, around 3.0% of preterm birth could be associated with GBS colonization (6*(1.21-1.00)*0.149/(1.21*0.149+1.00*0.851)/6.0 =0.18/6.0=3%).

Supplementary Table 3 is a frequency cross table of GBS colonisation and preterm/term birth in Japan.

**Supplementary Table 3: Frequency cross table of GBS colonisation and preterm/term birth in Japan**

|  | Preterm birth | Term birth |  |
| --- | --- | --- | --- |
| GBS colonisation + | a | c | 0.149* |
| GBS colonisation - | b | d | 0.851 |
|  | 0.056** | 0.94 | 1 |

* GBS colonization prevalence is reported to be 14.9% in Japan^16^

** Preterm birth rate is 5.6% in Japan^22^

Considering that RR is 1.21 for preterm birth if mother have GBS colonisation^23^

- $RR=\frac{\frac{a}{0.149}}{\frac{b}{0.851}}=1.21$
- $a+b=0.0566$

By solving the simultaneous equation,

$$a=0.009810489$$

Preterm birth attributable to GBS colonization could be calculated as

$$a*\frac{RR-1}{RR}=0.00170820$$

The percentage of this in overall preterm birth would be

$$\frac{0.00170820}{0.0566}=3.03\%$$

Information on newborns’ gestational age at delivery, and deaths, are reported by the Japan Perinatal Registry.^24^ This registry reports neonatal mortality before 28 weeks’ gestation and from 28 weeks’ gestation onward. Of all cases of preterm birth, 8.12% are before 28-weeks’ gestation. Parameter values regarding preterm birth used in the cost-effectiveness analysis are shown in Supplementary Table 4.

**Supplementary Table 4 Parameter values related to preterm birth in the cost-effectiveness analysis of GBS vaccination in Japan**

| **Variable** | **Value** | **Reference** |
| --- | --- | --- |
| GBS colonization prevalence | 14.9% | ^16^ |
| Preterm birth rate | 5.6% | ^22^ |
| Risk ratio of preterm birth for GBS positive mothers | 1.21 | ^23^ |
| Vaccine effectiveness to prevent GBS-related preterm birth | 50%, 80% | assumption |
| Proportion of <28w among preterm birth | 4.5% | ^22^ |
| Neonatal mortality of <28w (per 1000 live births) | 76.34 | ^24^ |
| Neonatal mortality of >=28w (per 1000 live births) | 1.25 | ^24^ |
| Risk difference of neonatal mortality | 75.09 |  |
| Cost per preterm case | ¥516,079 -1,232,280 | ^13,20^ |

## 9. Parameters and distributions used in probabilistic sensitivity analysis

Incidence, proportion of meningitis, sepsis and others and risk of death were modelled based on the national survey of infant GBS disease in Japan^4,25^ which reports actual numbers of cases, in each category, and outcomes (EOD/LOD, meningitis/sepsis/others, died/survived). For example, of 362 cases of GBS meningitis of term infants, 17 died and 345 survived according to the national surveys.^4,25^ So we modelled the distribution of probability of death from meningitis to be Beta (18, 346). For clinical syndrome, we used Dirichlet distribution. For example, the national survey reported 252 cases of EOD of term infants, of which 74 were meningitis, 175 were sepsis and 3 were other infections so proportions of meningitis, sepsis and other infections were parametrised according to Dirichlet (75, 176, 4). To estimate the range, sampling of 1,000 times were given and set of values were sorted by order of proportion of meningitis and range was taken from 2.5 % and 97.5% value. The same was done for proportion of sepsis and other infections.

## 10. References

1. Giorgakoudi, K. *et al.* Cost-effectiveness analysis of maternal immunisation against group B Streptococcus (GBS) disease: A modelling study. *Vaccine* **36**, 7033–7042 (2018).

2. Ishikawa, T. *et al.* Epidemiology of bacterial meningitis in children: Aichi prefecture, Japan, 1984-1993. *Pediatr. Neurol.* **14**, 244–250 (1996).

3. Kohli-Lynch, M. *et al.* Neurodevelopmental Impairment in Children after Group B Streptococcal Disease Worldwide: Systematic Review and Meta-analyses. *Clin. Infect. Dis.* **65**, S190–S199 (2017).

4. Matsubara, K. *et al.* Group B streptococcal disease in infants in the first year of life: a nationwide surveillance study in Japan, 2011–2015. *Infection* **45**, 449–458 (2017).

5. van der Ree, M., Tanis, J. C., Van Braeckel, K. N. J. A., Bos, A. F. & Roze, E. Functional impairments at school age of preterm born children with late-onset sepsis. *Early Hum. Dev.* **87**, 821–826 (2011).

6. Ministry of Health Labor and Welfare, J. Simple life table 2020. https://www.e-stat.go.jp/stat-search/files?page=1&layout=datalist&toukei=00450012&tstat=000001031336&cycle=7&tclass1=000001060864&tclass2=000001163166&tclass3val=0 (2021).

7. The Japan Council for Quality Health Care. Medical research report on prognosis of severe cerebral palsy. (2011).

8. Day, S. M., Reynolds, R. J. & Kush, S. J. Extrapolating published survival curves to obtain evidence-based estimates of life expectancy in cerebral palsy. *Dev. Med. Child Neurol.* **57**, 1105–18 (2015).

9. Akker-van Marle, M. E. *et al.* Cost-effectiveness of different treatment strategies with intrapartum antibiotic prophylaxis to prevent early-onset group B streptococcal disease. *BJOG An Int. J. Obstet. Gynaecol.* **112**, 820–826 (2005).

10. Oostenbrink, R., Moll, H. A. & Essink-Bot, M. L. The EQ-5D and the Health Utilities Index for permanent sequelae after meningitis: A head-to-head comparison. *J. Clin. Epidemiol.* **55**, 791–799 (2002).

11. Jarl, J., Alriksson-Schmidt, A. & Rodby-Bousquet, E. Health-related quality of life in adults with cerebral palsy living in Sweden and relation to demographic and disability-specific factors. *Disabil. Health J.* **12**, 460–466 (2019).

12. Carroll, A. E. & Downs, S. M. Improving Decision Analyses: Parent Preferences (Utility Values) for Pediatric Health Outcomes. *J. Pediatr.* **155**, 21-25.e5 (2009).

13. Ministry of Health Labor and Welfare, J. Fee of Medical Service Payment System 2020. https://clinicalsup.jp/contentlist/shinryo/ika/index.html (2020).

14. Honda, A., Nakao, H., Shoji, K., Kubota, M. & Ishiguro, A. Neonatal group B streptococcal ventriculitis without red flags for meningitis. *Pediatr. Int.* **62**, 996–998 (2020).

15. Ueda, N. K. *et al.* Neonatal meningitis and recurrent bacteremia with group B Streptococcus transmitted by own mother’s milk: A case report and review of previous cases. *Int. J. Infect. Dis.* **74**, 13–15 (2018).

16. Miyata, A. *et al.* Early-onset group B streptococcal disease following culture-based screening in Japan: A single center study. *J. Obstet. Gynaecol. Res.* **38**, 1052–1056 (2012).

17. Matsubara, K., Nigami, H., Harigaya, H., Yura, K. & Aoyagi, Y. [A case of group B streptococcal occult bacteremia]. *Kansenshogaku Zasshi.* **77**, 461–4 (2003).

18. Matsubara, K., Sugiyama, M., Hoshina, K., Mikamo, H. & Baba, K. Early onset neonatal sepsis caused by serotype VIII group B streptococci. *Pediatr. Infect. Dis. J.* **19**, 359–60 (2000).

19. Bacterial Meningitis Clinical Guideline 2014. *Bacterial Meningitis Clinical Guideline Committee, Japanese Society of Neurology, Japanese Society of Neurological Therapeutics, Japanese Society for Neuroloinfectious Diseases* (2014).

20. Ministry of Health Labor and Welfare, J. Medical Benefit Survey 2017. https://www.e-stat.go.jp/stat-search/files?page=1&layout=datalist&toukei=00450389&tstat=000001044924&cycle=0&tclass1=000001044945&tclass2=000001132104 (2019).

21. Ministry of Health Labor and Welfare, J. System of Nurse Visit. (2015).

22. Japanese Government. Population dynamics surveys Population dynamics statistics Exact number of births 4-24 Number and percentage of births in each year of gestation (4 weeks, early - early - late). *E-Stat: General contact point for government statistics* https://www.e-stat.go.jp/dbview?sid=0003411613 (2019).

23. Bianchi-Jassir, F. *et al.* Preterm Birth Associated with Group B Streptococcus Maternal Colonization Worldwide: Systematic Review and Meta-analyses. *Clin. Infect. Dis.* **65**, S133–S142 (2017).

24. Japan Society of Obstetrics and Gynecology, J. Perinatal statistics 2019. https://www.jsog.or.jp/modules/committee/index.php?content_id=8 (2020).

25. Shibata, M. *et al.* Epidemiology of group B streptococcal disease in infants younger than 1 year in Japan: a nationwide surveillance study 2016–2020. *Eur. J. Clin. Microbiol. Infect. Dis.* **41**, 559–571 (2022).
